# 

**DOI:** 10.1192/bjb.2017.31

**Published:** 2018-08

**Authors:** Martin Curtice

**Affiliations:** Consultant in Old Age Psychiatry, Worcestershire Health and Care NHS Trust, UK; email: mjrc68@doctors.org.uk

The Mental Capacity Act (MCA) has now been in operation for 10 years, and MCA case law has proliferated over this time. Books on this subject still remain vital to continue to embed this statute in practice. This book is a reprint of the original publication in 2013. It is aimed at psychiatrists and other mental health professionals and aims to provide ‘user-friendly’ guidance for medico-legal dilemmas that require an understanding of the MCA (and where it juxtaposes with the MHA statute). It is a readable book of only six chapters and 128 pages, which should appeal to busy professionals.

All chapters contain salient advice. Probably the most apposite chapter is that on best interests, especially with important case law having emerged in this area in recent years since the book was published (and given a prescient statement regarding best interests assessments and the need to fully involve the person and people who know them well that psychiatrists ‘have not been accustomed to this type of thinking’, which this chapter readily addresses). The section on the role of the Court of Protection is enlightening. Useful advice around the practical challenges of applying the MCA in the clinical setting is elucidated in the final chapter analysing clinical ambiguities in the assessment of capacity.

The book could have been improved for the reader by having easy to read learning points at the end of each chapter, and by having an annexe with the relevant MCA sections cited in full. Also, because case law does evolve, having a section recommending various legal resources would be helpful to enable professionals to readily keep up to date, e.g. online legal search engines and access to free monthly legal newsletters. A table comparing enduring power of attorney and both types of lasting power of attorney would also be useful.

It is unclear as to why the book has been reprinted without being updated with the advances in MCA case law since 2013. Some of these advances have been seminal in nature, especially in the areas of consent to treatment, best interests, end-of-life care and Do Not Attempt Cardiopulmonary Resuscitation orders, and, importantly Deprivation of Liberty Safeguards (DoLS), namely the ‘acid test’ that emerged from the Supreme Court after a ‘DoLS-athon’ in the lower courts. However, it still covers many areas of the MCA well enough for clinical practice, and is indeed ‘user-friendly’. It is a shame it wasn't updated, as it would undoubtedly then be an extremely useful book to have for clinical practice.

